# Risk Factors for Anastomotic Leak in Patients Undergoing Surgery for Rectal Cancer Resection: A Retrospective Analysis

**DOI:** 10.7759/cureus.79647

**Published:** 2025-02-25

**Authors:** Daniel Doniz Gomez Llanos, Carlos Alberto Leal Hidalgo, Sara Fernanda Arechavala Lopez, Alejandra Judith Padilla Flores, José Manuel Correa Rovelo, Amado De Jesús Athie Athie

**Affiliations:** 1 Surgery, Facultad Mexicana De Medicina, Universidad La Salle México, Mexico City, MEX; 2 Surgery, Hospital Médica Sur, Mexico City, MEX; 3 Surgery, Universidad La Salle México, Mexico City, MEX; 4 Medicine, Universidad Autónoma Metropolitana, Mexico City, MEX

**Keywords:** anastomotic leak, anterior resection, postoperative complication, rectal cancer, risk factors

## Abstract

Introduction

Anastomotic leakage (AL) is one of the most severe complications following rectal cancer (RC) surgery, with significant implications for morbidity, mortality, and oncological outcomes. Identifying risk factors associated with AL may enhance surgical decision-making and improve patient prognosis.

Methods

A retrospective cohort study was conducted, including 42 adult patients who underwent RC resection at a hospital in Mexico City between January 2015 and December 2022. Demographic, clinical, pathological, and surgical variables were analyzed to assess their association with AL. Univariate and multivariate statistical analyses were performed to identify independent risk factors.

Results

The overall incidence of AL was 11.9%, consistent with previous literature. Univariate analysis revealed no significant differences in patient-related factors such as age, BMI, ASA classification, diabetes mellitus, smoking, or biochemical markers (p>0.05). Treatment-related factors such as neoadjuvant therapy and diverting stoma (DS) placement did not show a significant association with AL. However, surgical factors played a crucial role: operative time was significantly longer in patients with AL (349.0 vs. 232.9 minutes, p=0.024), intraoperative blood loss was markedly higher (800.0 vs. 198.6 mL, p<0.001), and transfusion rates were elevated (60.0% vs. 13.5%, p=0.040). Tumor location in the middle rectum was more frequent among AL cases (60.0% vs. 18.9%, p=0.090). Postoperative complications were significantly more severe in patients with AL, with prolonged hospital stays (20.0 vs. 10.2 days, p=0.043) and increased reintervention rates (80.0% vs. 5.6%, p<0.001). In the logistic regression model, none of the analyzed variables reached statistical significance (p>0.99). However, operative time showed an odds ratio (OR) of 1.736 (p=0.997), suggesting that for each additional minute of surgery, the risk of AL could increase by 73.6%. Despite this trend, the wide confidence interval limits its precision and clinical applicability. Age showed an OR of 0.023 (p=0.998), potentially suggesting a 97.7% reduction in leakage risk for each additional year, although this result was not statistically significant and should be interpreted with caution.

Conclusion

Although no statistically significant risk factors were identified in the multivariate analysis, intraoperative variables such as prolonged surgical time, high blood loss, and transfusion requirement emerged as clinically relevant trends. These findings emphasize the need for optimizing surgical techniques and perioperative management to mitigate AL risk. Further studies with larger sample sizes are necessary to validate these associations and improve risk stratification models.

## Introduction

Colorectal cancer (CRC) is the third most common type of cancer worldwide, with approximately 1,926,425 new cases annually, and is the second leading cause of cancer-related death, with 904,019 associated deaths per year [1]. The disease accounts for 10% of all new cancer diagnoses, resulting in a significant health, economic, and social burden globally [2,3].

In the United States, it is the third most common cancer in both sexes and the third leading cause of death from this pathology [4,5]. In Mexico, it represents the third most frequent cancer for both men and women, with around 16,082 new cases in 2022 (7.8% of all diagnosed neoplasms) and sixth in the number of deaths. 30% of all colorectal neoplasms originate in the rectum [4]. According to GLOBOCAN statistics, rectal cancer (RC) ranks eighth in incidence worldwide, with 729,833 new cases, and tenth in mortality, with 343,817 deaths [6] and in Mexico it ranks sixteenth in frequency (1.7% of all neoplasms) and fifteenth in deaths (1.5%) [7].

The main modality and current cornerstone of treatment for RC is surgical resection. This can be preceded by neoadjuvant therapy such as chemoradiotherapy or adjuvant therapy with postoperative chemotherapy, considering multiple factors such as tumor size and location, clinical stage, and surgical procedure to be performed [4,8].

In the surgical management of RC with curative intent, the choice of surgical approach will depend on the tumor's height in relation to the anal margin (AM), the involvement of adjacent structures (levator ani muscles, seminal glands, and bladder), and the involvement of the anal sphincter complex. The main transabdominal surgical procedures used for the resection of RC, with subsequent primary anastomosis and sphincter preservation, are low anterior resection (LAR) with colorectal anastomosis for patients with tumors in the upper third of the rectum, and ultra-low anterior resection (ULAR) with coloanal anastomosis, with or without intersphincteric dissection, for patients with tumors in the middle or lower thirds of the rectum [4,9].

It is important to consider the potential postoperative adverse results that significantly increase morbidity and mortality in RC surgery procedures. Anastomotic leak (AL) is one of the most severe complications in patients undergoing RC surgery. The incidence of AL in this specific population ranges from 3% to 36.3% [4,10-14], with an estimated mortality rate of 6% to 30% [15] and a risk of permanent stoma of 10%-100% [14]. Its occurrence has been associated with increased local recurrence (LR), decreased quality of life, reduced long-term survival, and poor oncological outcomes [4,16,17].

Multiple studies in the literature have identified numerous risk factors associated with the development of AL after colorectal surgery and can be divided into patient-, disease- and treatment-related factors. Identifying those risk factors for AL can help surgeons use a tailored approach for decision-making, but in the present day, it is not possible to predict the occurrence of leakage for a specific patient.

In this study, we retrospectively analyzed cases of RC surgery performed at our hospital, examining the frequency of AL, and evaluated the clinical, surgical, and pathological characteristics to identify risk factors for AL in a surgical reference center in Mexico City.

## Materials and methods

A retrospective and observational analysis of clinical features, surgical data, and pathological characteristics was conducted, including 42 adult patients who underwent RC resection surgery at a tertiary care hospital in Mexico City from January 2015 to December 2022. All of the patient's data and variables were extracted from medical records, codified and de-identified to protect personal data and the patient's privacy, after approval by the institutional medical ethics committee. 

Staging procedures and inclusion criteria

Patients over 18 years old with a histological diagnosis of rectal adenocarcinoma who underwent RC resection surgery (LAR/ULAR) with primary colorectal or coloanal anastomosis were included in the present study. The population evaluated underwent a complete clinical evaluation, laboratory tests with complete blood cell count, serum chemistry, and carcinoembryonic antigen (CEA). In all the patients, a preoperative staging of the disease was performed, including colonoscopy with biopsy, abdominopelvic computed tomography (CT) scan, with chest X-ray or CT. In some patients, a PET-CT scan complemented the preoperative staging. Digital Rectal Examination (DRE), magnetic resonance imaging (MRI), and/or lower digestive endoscopy were performed to assess tumor height [4]. 

RC was defined as tumors with distal extension greater than 15 cm from the AM [18], and lesions were categorized as high (>10 to 15 cm), middle (>5 to 10 cm), or low (up to 5 cm).

Staging of the disease was made according to the pathologic classification (pTNM) of the 8th edition of the American Joint Committee on Cancer (AJCC). 

Neoadjuvant chemo-radiotherapy (NACRT) is widely used as part of multimodal treatment strategies in RC and is a standard treatment for locally advanced RC (LARC). Patients with RC who received NACRT and those who went straight forward to surgery were included in the present study. The main goal of this preoperative treatment is to reduce LR and improve disease-free survival (DFS) and overall survival (OS) after proctectomy [4]. Indications for neoadjuvant treatment included patients with LARC: those with T3-T4 tumors and/or node-positive disease [19], close or involved circumferential resection margin (CRM), lateral pelvic node involvement, or extramural venous invasion (EMVI), especially in the mid- and lower rectum.

Exclusion criteria

Patients with incomplete medical records, those under 18 years of age, and those undergoing surgical techniques outside the study protocol (e.g., non-restorative surgeries such as Hartmann's procedure, abdominoperineal resection, pelvic exenteration, and transanal procedures) were not included in the present study.

Study variables

Data collection encompassed demographic information, as well as patient-, treatment-, and disease-related variables, which were analyzed and are defined in Table 1.

**Table 1:** Clinical, surgical, and pathological variables reviewed and analyzed to identify risk factors for AL BMI: body mass index; ASA: American Society of Anesthesiologists; LAR: low anterior resection; ULAR: ultra-low anterior resection; AJCC: American Joint Committee on Cancer; T: tumor; N: nodule; M: metastasis

Patient related (clinical)	Treatment related (surgical)	Disease related (pathological)
Age	Type of surgery (LAR/ULAR)	Histopathological diagnosis
Sex	Surgical approach (open, laparoscopic, robotic)	Differentiation grade
BMI	Diverting stoma	Tumor size
Charlson comorbidity index	Type of suture (manual/mechanical)	Tumor location
Serum albumin	Operative time	Tumor distance to the anal verge
Serum hemoglobin	Intraoperative bleeding	Pathological T status
ASA score	Blood transfusion	Pathological N status
Diabetes	Multi-organic resection	M status
Tobacco use		Clinical stage (classification by the AJCC)
Neoadjuvant treatment		Lymph node harvest
Neoadjuvant chemo-radiotherapy		Number of lymph nodes positive for cancer
Time from neoadjuvant treatment to surgery		
Previous intra-abdominal surgery		

Definition and diagnosis of AL

AL was defined as an abnormal communication between the intraluminal and extraluminal compartments due to a defect in the integrity of the intestinal wall at the anastomosis site between the colon and rectum or colon and anus. Similarly, it can result from a leak at the site of manual or mechanical suture in a rectal reservoir or the presence of a pelvic abscess near the anastomosis site [20,21]. The findings that are considered as AL during re-operation are necrosis of the anastomosis, necrosis of a blind loop, dehiscence of the anastomosis, and signs of peritonitis [22].

The diagnosis is essentially clinical, with a wide range of manifestations such as tachycardia, abdominal pain, intolerance to oral intake, fever, distension, gas or fecal discharge through drains or surgical wounds, sepsis, and shock. It is complemented by laboratory tests (leukocytosis, elevated C-reactive protein (CRP), and elevated procalcitonin) and/or imaging studies such as oral or rectal contrast-enhanced tomography. Depending on its impact on clinical management, anastomotic leak is classified into three grades: Grade A (anastomotic leak that does not require active therapeutic intervention), Grade B (requires active therapeutic intervention without reoperation), and Grade C (requires reoperation) [20-22]. Treatment was defined based on variables such as clinical presentation, symptoms, hemodynamic stability, and presence of peritonitis and/or sepsis, and may range from fasting, intravenous antibiotic therapy, percutaneous drainage of collections, or surgical reoperation with remodeling or dismantling of the anastomosis, with the creation of a temporary or permanent stoma. 

Main outcomes

Patients were classified into two groups: patients with AL and patients without AL. The primary endpoint of the study was the detection of independent risk factors for AL in our study population. Secondary endpoints include the overall rate of anastomotic leak, surgical reintervention (defined as reintervention within 30 days after the primary operation), prolonged hospital stays, postoperative morbidity (assessed by the Clavien-Dindo classification (CDC) and by the Comprehensive Complication Index (CCI®)), and mortality.

Morbidity evaluation

For the analysis of this specific endpoint, we used the CDC and the CCI®. In 2004, the introduction of the CDC [23] offered a standardized approach to rank complications in severity in a simple, intuitive, and reproducible way (Table 2). It is focused on reporting the single most severe complication experienced by a patient, sometimes ignoring events of lesser severity that contribute to overall morbidity developed from a surgical procedure.

**Table 2:** CDC CDC: Clavien-Dindo classification; CNS: central nervous system; IMC: intermediate care; ICU: intensive care unit

Grade	Definition
Grade 1	Any deviation from the normal postoperative course without the need for pharmacological treatment or surgical, endoscopic, and radiologic interventions. Allowed therapeutic regimens are as follows: drugs such as antiemetics, antipyretics, analgesics, diuretics, electrolytes, and physiotherapy. This grade also includes wound infections opened at the bedside
Grade 2	Requiring pharmacological treatment with drugs other than those allowed for grade I complications. Blood transfusions and total parenteral nutrition are also included
Grade 3	Requiring surgical, endoscopic, or radiological intervention
3a	Intervention not under general anesthesia
3b	Intervention under general anesthesia
Grade 4	Life-threatening complication (including CNS complications)* requiring IMC/ICU management
4a	Single-organ dysfunction (including dialysis)
4b	Multiorgan dysfunction
Grade 5	Death of a patient
Suffix “d”	If the patient suffers from a complication at the time of the discharge, the suffix “d” (for “disability”) is added to the respective grade of complication. This label indicates the need for a follow-up to fully evaluate the complication
*Brain hemorrhage, ischemic stroke, and subarachnoid bleeding, but excluding transient ischemic attacks.	

To overcome this obstacle, the CCI® was developed as a unique and novel tool for assessing the overall cumulative morbidity experienced by an individual patient, with scores from 0 (uneventful postoperative course) to 100 (death of the patient). The CCI® can be calculated with the specific recording of the grades of all complications in a patient, using the CDC as the complication reporting definitions [24,25]. Studies have demonstrated that the CCI® is significantly more sensitive than the CDC and other metrics in detecting treatment effects, requiring a smaller sample size in RCTs and best suited as a primary endpoint in surgical research [25). CCI® calculator is available at: https://www.cci-calculator.com/. Using both CDC and CCI® improves complication reporting and improves quality control, benefiting all healthcare participants and patients [24].

Statistical analysis

The Shapiro-Wilk test was used to assess the normality of continuous variables. Variables with normal distribution were analyzed using the Student's t-test for independent samples, while those with non-normal distribution were evaluated using the Mann-Whitney U test. Categorical variables were analyzed with Pearson’s chi-square test or Fisher's exact test, and Phi and Cramer’s V coefficients were calculated to determine the strength of associations (weak, moderate, or strong). Statistical significance was defined as p<0.05.

Finally, binary logistic regression analysis was performed using a forward stepwise method based on partial likelihood ratios to identify risk factors associated with AL. The model was adjusted using the maximum likelihood estimation method, reporting coefficients (B), odds ratios (OR), 95% confidence intervals (CI), and p-values. Statistical analyses were conducted using IBM® SPSS Statistics version 29.0 (IBM Corp., Armonk, US).

## Results

Data from 42 patients with a mean age of 63.0 years (21 males and 21 females) were analyzed. Studied factors were classified into patient-, disease- and treatment-related factors. 

In the univariate analysis, relevant differences were identified between patients with and without AL. Demographic and patient-related factors are shown in Table 3. No significant differences were observed in the mean age of patients (57.0 vs. 63.8 years, p=0.296). Patients with AL had a higher body mass index (BMI) (26.6 vs. 24.0 kg/m², p=0.104). A higher proportion of patients classified as ASA III was found in the leakage group (400% vs. 16.2%, p=0.080). Neither the Charlson Comorbidity Index (p=0.758), smoking (p=0.637), nor diabetes mellitus (p=0.213) showed a significant association with leakage. In terms of biochemical parameters, both preoperative albumin levels (3.6 vs. 3.5 g/dL, p=0.822) and preoperative hemoglobin levels (12.7 vs. 13.1 g/dL, p=0.632) showed no significant differences between the groups. Twenty-two patients (52.38%) received neoadjuvant treatment, all of whom received chemotherapy (100%), and 19 received radiotherapy (86.36%). The mean time from the end of neoadjuvant treatment to surgery was 59 days, with no significant difference between the groups (63.6 vs. 58.95 days, p=0.806).

**Table 3:** Patient-related factors at the time of admission The p-value <0.05 is considered statistically significant. BMI: body mass index; ASA: American Society of Anesthesiologists

Variable		Presence of leakage (n=5)	No leakage (N=37)	p-value
Demographic and anthropometric factors				
Age (years)		57.0 (±6.0 SE)	63.8 (±2.2 SE)	0.296
Sex (N%)	Woman	1 (20.0%)	20 (54.1%)	0.343
	Man	4 (80.0%)	17 (40.5%)	
BMI (Kg/m^2^)		26.6 (±1.8 SE)	24.0 (±0.5 SE)	0.104
BMI classification (N%)	Low weight	1 (20.0%)	3 (8.1%)	0.060
	Normal weight	1 (20.0%)	20 (54.1%)	
	Overweight	1 (20.0%)	12 (32.4%)	
	Obesity	2 (40.0%)	2 (5.4%)	
Diabetes (N%)		0 (0.0%)	9 (24.3%)	0.213
Smoking (N%)		3 (60.0%)	11 (29.7%)	0.637
Preoperative clinical factors				
ASA physical status (N%)	I	1 (20.0%)	1 (2.7%)	0.080
	II	2 (40.0%)	30 (81.1%)	
	III	2 (40.0%)	6 (16.2%)	
	IV	0 (0.0%)	0 (0.0%)	
Charlson comorbidity index (points)		4.6 (±1.3 SE)	4.9 (±0.3 SE)	0.758
Serum albumin (g/dL)		3.6 (±0.2 ES)	3.5 (±0.1 SE)	0.822
Serum hemoglobin (mg/dL)		12.7 (±0.4 SE)	13.1 (±0.2 SE)	0.632
Neoadjuvant chemotherapy (N%)		3 (60.0%)	19 (51.4%)	0.716
Neoadjuvant radiotherapy (N%)	No radiotherapy	1 (20.0%)	2 (5.4%)	0.647
	Short course	0 (0.0%)	2 (5.4%)	
	Long course	2 (40.0%)	15 (40.5%)	
Time from neoadjuvant treatment to surgery (days)		62.6 (±18.9 SE)	58.95 (±5.2 SE)	0.806
Previous abdominal surgery (N%)		1 (20.0%)	18 (48.6%)	0.227

Treatment-related factors are shown in Table 4. Twenty-four patients (57.14%) underwent LAR and 18 (42.85%) underwent ULAR, with a mean operating time of 246 minutes and intraoperative blood loss of 270 mL. A diverting loop ileostomy was performed in 28 (66.66%) patients. Four of five patients (80%) with AL had a protective stoma, with no difference in the loop ileostomy construction in preventing AL (p=0.500). The surgical approach adopted was open approach (59.52%), laparoscopic approach (35.71%), and robotic (4.76%), with no difference in AL incidence (open surgery 12%, laparoscopic surgery (LS) 13.33%, p=0.861).

**Table 4:** Treatment-related factors The p-value <0.05 is considered statistically significant. LAR: low anterior resection; ULAR: ultra-low anterior resection

Variable		Presence of leakage (n=5)	No leakage (N=37)	p-value
Intraoperative factors				
Type of surgery (N%)	LAR	2 (40.0%)	22 (59.5%)	0.409
	ULAR	3 (60.0%)	15 (40.5%)	
Approach (N%)	Laparoscopic	2 (40.0%)	13 (35.1%)	0.861
	Open	3 (60.0%)	22 (59.5%)	
	Robotic	0 (0.0%)	2 (5.4%)	
Protective stoma (N%)		4 (80.0%)	24 (64.9%)	0.500
Type of anastomosis (N%)	Manual	2 (40.0%)	7 (18.9%)	0.288
	Mechanical	3 (60.0%)	30 (81.1%)	
Surgical time (min)		349.0 (±50.6 SE)	232.9 (±16.8 SE)	0.024
Transoperative bleeding (mL)		800.0 (±348.2 SE)	198.6 (±160.5 SE)	0.000
Intra-/postoperative blood transfusion (N%)		3 (60.0%)	5 (13.5%)	0.040
Resection of other structures/organs (N%)		3 (60.0%)	7 (18.9%)	0.078

Significant differences were observed, highlighting the impact of surgical techniques on outcomes. Surgical time was significantly longer in patients with leakage (349.0 vs. 232.9 minutes, p=0.024), intraoperative bleeding was significantly higher in the leakage group (800.0 vs. 198.6 mL, p<0.001), and the need for intra- and/or postoperative transfusion was more frequent in the leakage group (60.0% vs. 13.5%, p=0.040). On the other hand, factors such as anastomotic technique (manual vs. mechanical, p=0.288) or multi-organ resection (p=0.078) did not show significant differences.

Disease-related factors are shown in Table 5. No significant differences were found in mean tumor size (3.2 vs. 3.1 cm, p=0.975). Tumor location showed an interesting trend: tumors in the middle third of the rectum were more frequent in patients with leakage (60.0% vs. 18.9%, p=0.090). Tumor height in relation to the AM showed no differences between groups (12.1 vs. 9.5 cm, p=0.243). 

**Table 5:** Disease-related factors The p-value <0.05 is considered statistically significant. G: grade; T: tumor; N: nodule; M: metastasis; pT: pathological T; pN: pathological N; pCR: complete pathological response

Variables				Presence of leakage (n=5)	No leakage (N=37)	p-value
Tumor factors						
Tumor location (N%)	Upper third			2 (40.0%)	18 (48.6%)	0.090
	Middle third			3 (60.0%)	7 (18.9%)	
	Lower third			0 (0.0%)	12 (32.4%)	
Tumor distance to anal margin (cm)				12.1 (±3.5 SE)	9.5 (±4.6 SE)	0.243
Tumor size (cm)				3.2 (±1.0 SE)	3.1 (±1.6 SE)	0.975
Degree of tumor differentiation (N%)		Well differentiated (G1)		0 (0.0%)	1 (2.7%)	0.769
		Moderately differentiated (G2)		5 (71.4%)	30 (81.1%)	
		Poorly differentiated (G3)		0 (0.0%)	2 (5.4%)	
		In the differentiated (G4)		0 (0.0%)	4 (10.8%)	
TNM staging (N%)	pT		T0	0 (0.0%)	5 (13.5%)	0.354
			Tis	1 (20.0%)	2 (5.4%)	
			T1	0 (0.0%)	4 (10.8%)	
			T2	0 (0.0%)	8 (21.6%)	
			T3	4 (80.0%)	15 (40.5%)	
			T4a	0 (0.0%)	3 (8.1%)	
			T4b	0 (0.0%)	0 (0.0%)	
	pN		N0	1 (20.0%)	23 (62.2%)	0.108
			N1a	1 (20.0%)	2 (5.4%)	
			N1b	1 (20.0%)	5 (13.5%)	
			N1c	0 (0.0%)	3 (8.1%)	
			N2a	2 (40.0%)	2 (5.4%)	
			N2b	0 (0.0%)	2 (5.4%)	
	M		M0	3 (60.0%)	32 (86.5%)	0.097
			M1a	0 (0.0%)	2 (5.4%)	
			M1b	2 (40.0%)	2 (5.4%)	
			M1c	0 (0.0%)	1 (2.7%)	
Clinical stage (N%)		pCR		0 (0.0%)	5 (13.5%)	0.350
		0		1 (20.0%)	2 (5.4%)	
		I		0 (0.0%)	10 (27.0%)	
		IIA		0 (0.0%)	4 (10.8%)	
		IIB		0 (0.0%)	1 (2.7%)	
		IIC		0 (0.0%)	0 (0.0%)	
		IIIA		0 (0.0%)	2 (5.4%)	
		IIIB		1 (20.0%)	7 (18.9%)	
		IIIC		1 (20.0%)	1 (2.7%)	
		IV		2 (20.0%)	5 (13.5%)	
Lymph node harvest (number of nodes)				16.6 (±4.9 SE)	15.6 (±7.8 SE)	0.788
Lymph nodes with neoplasia (number of nodes)				3.0 (±2.5 SE)	1.1 (±2.2 SE)	0.970

Although not significant, tumor staging showed a slight predominance of advanced stages in patients with leakage. Regarding the clinical stage, four of five patients with AL had a pathological TNM stage ≥IIIa (p=0.350). Lymph node (LN) harvest (16.6 vs. 15.6 LN, p=0.788) and cancer-positive LNs (3.0 vs. 1.1 LN, p=0.97) showed no difference between groups. 

Postoperative outcomes are shown in Table 6. Regarding postoperative outcomes, a significant impact of leakage on the patient's clinical evolution was evident. Hospital stay was considerably longer in the leakage group (20.0 vs. 10.2 days, p=0.043). Moreover, the rate of surgical reintervention was drastically higher in patients with leakage (80.0% vs. 5.6%, p<0.001), underscoring the severity of this complication. Patients with AL had higher CDC scores (p=0.000) and higher overall morbidity according to the CCI® (20.4 vs. 73.3, p=0.000).

**Table 6:** Postoperative outcomes The p-value <0.05 is considered statistically significant. CCI: comprehensive complication index

Variables		Presence of leakage (n=5)	No leakage (N=37)	p-value
Postoperative outcomes				
Days of hospital stay (days)		20.0 (±5.6 SE)	10.2 (±1.5 SE)	0.043
Surgical reintervention (N%)		4 (80.0%)	2 (5.6%)	0.000
CCI (N%)		73.3 (±27.5)	20.4 (±15.7)	0.000
Clavien-Dindo	No complication	0 (0.0%)	3 (8.1%)	0.000
	1	0 (0.0%)	21 (56.8%)	
	2	1(20.0%)	10 (27.0%)	
	3a	0 (0.0%)	1 (2.7%)	
	3b	1 (20.0%)	2 (5.4%)	
	4a	1 (20.0%)	0 (0.0%)	
	4b	1 (20.0%)	0 (0.0%)	
	5	1 (20.0%)	0 (0.0%)	
Mortality (N%)		1 (20.0%)	0 (0.0%)	0.119

In the logistic regression model designed to identify risk factors associated with AL, none of the analyzed variables reached statistical significance (p>0.99). However, certain clinical trends warrant consideration. Operative time showed an OR of 1.736 (p=0.997), suggesting that for each additional minute of surgery, the risk of leakage could increase by 73.6%. Nevertheless, the wide confidence interval limits the precision and clinical applicability of this finding. It is important to note that this result reflects a relative increase in the probability of presenting the event compared to not presenting it and not a direct proportional increase in the absolute risk. The interpretation of this finding should consider that surgical time was analyzed in units of minutes, which may amplify the effect observed when calculating the OR. This relationship, although not statistically significant, highlights the need to cautiously evaluate the association between procedure duration and risk of leakage, especially in future studies with larger sample sizes. Age demonstrated an OR of 0.023 (p=0.998), potentially indicating a 97.7% reduction in leakage risk for each additional year. While intriguing, this result lacks statistical significance and should be interpreted with caution.

## Discussion

AL is one of the most serious postoperative complications after RC surgery. It occurs despite developments in surgical technique and perioperative care. It is associated with prolonged hospital stay, reintervention, a stage-dependent decrease in survival, bowel dysfunction, and a high risk of permanent stoma [26]. AL is also associated with a greater risk of LR and poorer overall and cancer-specific survival [27]. 

The present analysis reported an AL incidence of 11.9%, which is consistent with previously published data. AL was diagnosed postoperatively at a median of 7.4 days (range: 2-14 days). In comparison, Jorgren et al. [28] reported a median onset of 12 days (range three to 30 days). Type C AL was identified in four patients (80%), while Type B AL occurred in one patient (20%). All patients with Type C AL required surgical intervention, which involved anastomosis takedown and the creation of an end stoma. The overall mortality rate was 2.5%, directly attributed to AL (20% mortality in the AL group). This finding aligns with previously reported mortality rates for AL related to CRC, which are described as 6% and 30% [15].

Risk assessment for this important complication is necessary, and an early decision-making algorithm must consider multiple factors in order to detect potential risks for AL presentation. We have found results that do not completely match with the current literature.

In the patient-related factors analyzed in the present study, age was not detected as a risk factor for AL (57.0 vs. 63.8.0 years, p=0.296). The effect of age on AL after CRC resection remains unclear. Brisinda et al. [14] reported that age >65 years is considered a significant risk factor for postoperative complications in RC surgery. A series of 1391 patients undergoing rectal surgery suggested that age over 60 years remains an independent risk factor for AL (HR 2.42) [29]. On the other side, a retrospective population-based cohort of 45,488 patients conducted by Zaimi et al. [30] showed a protective effect of age on colorectal AL. The incidence of AL was lowest in patients >80 years old, but older age was associated with higher mortality after AL. For patients >80 years old, the mortality posterior to AL was as high as 27.0%. 

Male gender has been previously reported as a risk factor for AL [15,16,31] and it may be explained by multiple factors, such as the narrow male pelvis, which makes dissection technically more difficult, and differences in intestinal microcirculation [32]. The present analysis showed that in the AL group, four of five patients were male (80%), but this value did not reach significance (p=0.343). 

In our study, patients with AL had a higher BMI (26.6 vs. 24.0 kg/m², p=0.104), suggesting that being overweight might influence the technical complexity of the surgical procedure and healing of the anastomosis. Studies made by McDermott et al. [11], Frasson et al. [33], and Nikolian et al. [34] have shown obesity to increase the risk of AL. Obesity is associated with an increased risk of anastomotic failure, especially in patients undergoing low rectal anastomosis. Interestingly, recent research also revealed that a BMI >30 kg/m^2^ was considered an independent risk factor for AL [35]. In a meta-analysis by Yang et al. [36], visceral adiposity was found to be related to a higher conversion rate to open surgery, prolonged operative times, AL, and higher morbidity rates.

Although not significant, a higher proportion of patients classified as ASA III was found in the leakage group (60.0% vs. 27.0%, p=0.109), reflecting a potential impact of comorbidity severity on surgical risk. The ASA score is a classification system used to evaluate a patient's overall health and is an effective tool for perioperative risk assessment. A retrospective analysis of 505 patients with colorectal resection and primary anastomosis revealed that the ASA score was found to be independently correlated with the development of AL and that a higher ASA score was significantly associated with AL (OR 2.99, 95% CI 1.345-6.670, p=0.007) [37].

Regarding preoperative serum albumin, we observed no differences in preoperative serum albumin levels and the risk of AL (3.6 g/dL vs. 3.5 g/dL, p=0.822). It has been studied and described that low preoperative levels of serum albumin [14,35] (<3.5g/dL) appear to be associated with an increased AL risk in patients suffering from various colorectal diseases, especially CRC and IBD (OR 2.8, 95% CI, 1.3-5.1, p=0.03) [38]. 

Hemoglobin is related to perfusion and oxygenation of the anastomotic borders, indispensable factors for anastomotic healing. According to Hayden et al. [39], a hemoglobin level lower than 11 g/dL increased the risk of AL 6.5-fold, explained by a decreased capacity to transport oxygen to the adjacent structures and a subsequent risk of ischemia. Brisinda et al. described a higher incidence of AL in patients with low hemoglobin levels (11.8% vs. 7.0% in level ≥10 g/dL, p=0.02) [14]. We did not find differences in the incidence of AL between both groups (12.7 g/dL vs. 13.1 g/dL, p=0.632). 

NACRT is widely used as part of multimodal treatment strategies in RC and is a standard treatment for LARC. In our study population, 22 (52.38%) patients were treated prior to surgery with chemo- and/or radiotherapy, and 20 patients (47.61%) went straight forward to surgical resection of RC. All of the 22 patients with NACRT (100%) were treated with chemotherapy and 19 of the 22 patients had concomitant radiotherapy. Two patients were treated with short-course radiotherapy (SCRT) and 17 patients received long-course radiotherapy (LCRT). Two patients who received LCRT+chemotherapy presented AL (two of 17 patients, 11.76%). In the present analysis, NACRT was not associated with AL.

Recent research relating to NACRT and the incidence of postoperative AL has shown variable results. Park et al. [15] developed a retrospective study on 1609 RC cancer patients after LS. The group found that NACRT was associated with an increased risk of AL in patients without protective stoma (HR 6.284, 95% CI, 2.829-13.961, p<0.001). Another study, by Hamabe et. al [40], analyzed 296 cases with RC who underwent laparoscopic LAR and found that neoadjuvant chemotherapy was associated with an elevated risk for AL (P=0.0222). Results from a large cross-sectional study also showed that NA for RC patients was an independent risk factor for developing AL during follow-up (OR 2.85, 95% CI, 1.00-8.11) [41]. This finding may be due to the local effects of radiation on the tissues as well as represent a marker of the location of the tumor. Most LARCs located in the mid and lower rectum receive neoadjuvant radiation. Also, radiation has been associated with decreased oxygen delivery to the tissues and impaired healing [39]. On the other hand, various researchers have supported that NACRT does not influence the development of AL. Brisinda et al. [14] found that NA therapy was not found to be associated with AL. The Medical Research Council (MRC) CR07 RCT [42] and a retrospective study of 1437 patients described by Chang et al. [43] concluded similar results. A meta-analysis from 1980 to 2015 developed by Hu et al. [44] demonstrated that NA therapy does not increase the incidence of AL after LAR for mid and low RC. In addition, neither the interval to surgery after NA therapy nor the RT regimen increased the AL.

Regarding the specific time for surgery after receiving NACRT, we found no differences between the compared groups. The mean time from the end of NACRT to surgery was 59 days (p=0.806). The Stockholm III randomized trial [45] compared SCRT 5×5 Gy + immediate surgery vs. SCRT 5×5 Gy with delayed surgery for four to eight weeks. The rate of postoperative complications was significantly lower in the delayed surgery group, and the AL rate was 11.8% vs. 7.2% (p=0.01). The meta-analysis conducted by Hu et al. [44], on the contrary, found that the interval to surgery after NA therapy does not increase the AL rate.

In the treatment-related factors analyzed, 24 patients (57.14%) underwent LAR and 18 (42.85%) ULAR. Three of 18 patients in the ULAR group (16.66%) and two of 24 patients in the LAR group (8.33%) developed an AL. A diverting loop ileostomy was performed in 28 (66.66%) patients. Four of five patients (80%) with AL had a protective stoma, with no difference in the loop ileostomy construction in preventing AL (p=0.500).

Operative time is a factor that impacts the prognosis of wound healing at the anastomotic site, and a longer surgical time might increase the risk of bacterial exposure and tissue impairment, which may cause inflammation. In the present study, the mean operating time was 246 minutes. We found that surgical time was significantly longer in patients with leakage (349.0 vs. 232.9 minutes, p=0.024), which could reflect the technical complexity of the procedure in these cases. Multiple studies in the literature match this finding. Brisinda et al. [14] found that the mean duration of surgery was longer in patients who developed AL (186.0±40.2 min) than in patients without AL (115.0±47.8 min, P=0.0001). Midura et al. [46] made a retrospective analysis of patients who underwent segmental colectomy with anastomosis, which included 13,684 patients. One of the factors that they found associated with AL on the multivariate logistic regression analysis was an operative time >3 hours (OR 1.5, 95% CI 1.19-1.9, p=0.001). Shiwakoti et al. [47] made a retrospective evaluation of 185 patients with RC who underwent laparoscopic LAR. It was reported in their study that prolonged surgical time ≥180 minutes was an independent risk factor related to AL (OR 2.476, 95% CI 1.033-5.932, p=0.042). Similar results were obtained by Kim et al. [48]. Nevertheless, in our study, the obtained results were not found to be independently predictive of AL on the multivariate analysis.

In our review, the mean intraoperative blood loss was 270 mL. Intraoperative bleeding was significantly higher in the leakage group (800.0 vs. 198.6 mL, p<0.001), suggesting that procedures with greater blood loss might compromise anastomotic healing. Telem et al. [38] in 2010 reported that intraoperative blood loss of 200 mL or more increased the risk of AL presentation (OR 3.1, 95% CI, 1.9-5.3, p=0.01). A similar difference was found for intraoperative blood loss (365.0±50.0 mL in patients with AL vs. 175.5±45.0 mL in patients without AL, p=0.000) in the study performed by Brisinda et al. [14]. Reviewing risk factors for AL in laparoscopic LAR for RC, Kawada et al. pointed out that blood loss greater than 100 mL and blood transfusion are independent risk factors for AL, being unclear whether this is a specific manifestation due to blood loss or whether blood loss is an indirect indicator for challenging surgery or deficient operative technique [12]. Blood loss during surgery ≥400 mL was also reported as an independent factor for relaparotomy in patients with AL in a study performed by Zhang et al. [49].

The need for intra and/or postoperative transfusion was more frequent in the leakage group (60.0% vs. 13.5%, p=0.040), which could be related to the adverse effects of hemodilution, a more complicated or difficult procedure, or a poorer overall condition of the patient. Brisinda et al. [14] showed that a higher incidence of AL was observed in patients receiving blood transfusions (14.8%) compared to those who did not require this therapy (6.8%, P=0.002). Similar results were demonstrated by Kawada et al. [12], Park et al. [15], and Xu & Kong [50]. 

The surgical approach performed in the studied population was open surgery (59.52%), LS (35.71%), and robotic surgery (RS) (4.76%), with no difference in AL incidence (open surgery 12% vs. LS 13.33%, p=0.861). Multiple studies have been conducted to compare results between open vs. minimally invasive surgery (MIS) techniques.

The five-year follow-up of the CLASICC trial [51] was set up to evaluate the technical and oncological efficacy and safety of LS in comparison with open surgery for the treatment of CRC. It investigated the five-year outcomes: OS, DFS, locoregional (LRR), wound/port-site, and distant recurrences. For RC, the five-year OS rate was 52.9% for open vs. 60.3% for LS (p=0.132) and the five-year DFS rate was 52.1% for open vs. 53.2% for LS (p=0.953). For anterior resection, the five-year OS rate was 56.7% for open vs. 62.8% for LS (p=0.247).

A Cochrane review of 10 RCTs including 2505 patients [52] comparing laparoscopic vs. open TME for RC showed similar OS and DFS, as well as similar tumor recurrences. Operative times were shorter with the open procedure, and the LS approaches had decreased blood loss and shorter hospital stays. The incidence of AL was not different between groups. Long-term results of the COLOR II Study Group [53] included 1044 patients and aimed to study LRR recurrence three years after index surgery, DFS, and OS in patients with RC, comparing open vs. laparoscopic approach. LS in patients with RC was associated with rates of LRR, DFS, and OS similar to those for open surgery. There were no significant differences in the rates of AL, complication, or death. 

Kim et al. [54] reviewed and compared risk factors for AL after LAR for RC between MIS and open surgery in 1704 patients who underwent elective LAR with colorectal anastomosis for RC. The overall incidence of AL was similar between groups (5.8% in OS, 7.3% in LS, 5.4% in RS, p=0.345). Risk factors for AL in the MIS group were more related to technical difficulties such as male sex, previous abdominal surgery, operation time >200 minutes, lower location of the tumor, and use of more than one stapler for distal rectal resection.

Regarding the type of anastomosis made (stapled vs. hand-sewn), our study reported 33 patients with stapled anastomosis (78.57%) and nine hand-sewn anastomosis (21.42%). In the AL group (five patients), three (60%) had stapled anastomosis and two (40%) a hand-sewn anastomosis. No statistical difference was presented between both groups (p=0.288). This result is consistent with the actual literature. A Cochrane review by Neutzling et al. [55] compared the use of suture vs. stapling devices for anterior resection and found that neither technique was superior to the other in terms of AL rate. This result is shared by the data obtained from an analysis made by Arezzo et al. [16], which indicated equal AL rates with stapled and hand-sewn anastomoses, and a more recent meta-analysis presented by Oliveira et al. [56] showed similar outcomes.

In the present study, a diverting loop ileostomy was performed in 28 (66.66%) of the 42 patients. Four of five patients (80%) with AL had a protective stoma, with no difference in the loop ileostomy construction in preventing AL (p=0.500). The creation of a diverting stoma (DS) and its role in preventing AL and protecting anastomosis has been a controversial topic in the literature. Construction of a DS is often temporary with an intent to protect a downstream anastomosis by keeping the area clean from stool passage, with the objective of diminishing rates of clinically evident colorectal AL and decreasing severe complications and reoperation rates [57]. 

Wong et al. [58] developed a study of 1066 patients who underwent elective LAR/ULAR in a 10-year period, aiming to determine if a DS is really necessary after a low anastomosis. The conclusion was that a DS does not reduce postoperative AL (p=0.8633), but it reduces the catastrophic effects of an AL. A multicenter RCT with 234 patients developed by Matthiessen et al. [59] assessed the rate of AL in patients operated with LAR for RC randomized to a DS or not. The overall rate of AL was 19.2% and patients without a stoma had an AL rate of 28% (vs. DS 10.3%, OR 3.4, 95% CI, 1.6-6.9, p≤0.001) and the need for urgent reoperation was higher in those with no DS (25.4% vs. 8.6%, p<0.001), concluding that DS decreased the rate of symptomatic AL and recommending its use in LAR for RC, results supported by Shiomi et al. [60]. Zhang et al. indicated that a DS should be performed in sphincter-preserving surgery for middle and low RC patients with two or more risk factors identified in their analysis (male sex, diabetes mellitus, K-RAS mutation, distance of the tumor from the anal verge (AV), and NACRT), which were independent risk factors for AL [49].

Kawai et al. [61] analyzed the risks and benefits of DS creation during RC surgery, including 400 participants and dividing them into DS (+) and DS (-) groups, and compared the postoperative complications and outcomes between them. Overall AL was similar between groups and DS helped prevent severe peritonitis related to AL. Also, they reported that patients with DS developed more postoperative complications (stoma outlet syndrome, ileus, and bowel obstruction) and prolonged hospital stays. A recent meta-analysis of nine RCTs demonstrated that patients in the DS group had lower odds of AL (OR 0.362, 95% CI, 0.236-0.555, p<0.001), complications (OR 0.61, 95% CI 0.461-0.828, p<0.001), abscess (OR 0.392, 95% CI 0.174-0.883, p<0.024), and reoperation (OR 0.352, 95% CI 0.222-0.559, p<0.001) than the no-diversion group [62]. 

Regarding disease-related factors, the present study found that rectal tumor location was divided into upper rectum (47.61%), middle rectum (23.8%), and distal rectum (28.57%). Tumor location showed an interesting trend: tumors in the middle rectum were more frequently associated with AL (60.0% vs. 18.9%, p=0.090). This may be due to specific anatomical characteristics, such as a more difficult dissection during surgery and the clinical stage of the cancer treated. 

The mean tumor distance to the AV was 9.9 cm, with no difference in AL incidence depending on the AV distance to the inferior margin of the tumor (12.1 vs. 9.5 cm, p=0.243). This result differs from literature reports. Tumor distance from the AV is a well-studied and recognized risk factor for the development of AL [11,39,49,61]. A study of 35 patients demonstrated that resection of rectal tumors 12 cm or less from the AV conferred an increased incidence of anastomotic dehiscence compared with tumor resection >12 cm away (7.4% vs. 3.0%) (OR, 4.5) [38]. Brisinda et al. [14] identified that tumor distance from the AV was a risk factor for AL (71.0±32.0 mm in AL patients vs. 89.0±21 mm in patients without AL, p=0.0001). 

The mean tumor size in the present study was 3.3 cm. Tumor size is a tumor-related factor associated with the development of AL according to McDermott et al. [11] and Kawada et al. [12]. A possible explanation for this finding is that the pelvic space is limited in patients with larger tumors, which can complicate the ease of rectal transection and anastomosis. This results in a more difficult mobilization of large tumors with increased tissue tension and compromised microcirculation, especially in patients with comorbidities [47]. Shiwakoti et al. [47] found that a tumor size ≥5 cm (23.40%) was associated with a higher risk of AL than was a smaller tumor size of <5 cm (14.65%) and Brisinda et al. [14] reported that an RC diameter greater than 3 cm was identified as an independent risk factor for AL. We found no difference in tumor size regarding AL (AL 3.2 cm vs. 3.1 cm, p=0.975). 

Analyzing the pathological TNM staging (pTNM) in our population, we observed that four patients with a pT3 (80%) presented AL (p=0.354). In the pN staging, one N1a (20%), one N1b, and two N2a patients (40%) presented AL (p=0.108). Related to the M staging, three patients without metastasis (60%) and two M1b (40%) presented AL (P=0.097). Although not significant, tumor staging showed a slight predominance of advanced stages (one patient in clinical stage IIIB, one patient in clinical stage IIIC, and two patients in clinical stage IV) in patients with AL (p=0.350). This trend is consistent with previous studies [14,50] and it may be explained by the more technical complexity of such cases and the overall clinical status of the patient [16]. Five patients obtained a pathological complete response (pCR) after NACRT (22.72%) and surgical resection, with no AL developed in that group. LN harvest (16.6 vs. 15.6, p=0.788) and cancer-positive LNs (3.0 vs. 1.1, p=0.97) showed no difference between groups for AL.

With reference to the postoperative outcomes, we observed a significant increase in the mean postoperative hospital stay (10.2 vs. 20.0 days, p=0.043) and the incidence of severe complications (patients with AL had higher Clavien-Dindo scores (p=0.000) and overall morbidity, calculated by the CCI®, 20.4±15.7 vs. 73.3±27.5 in the AL group, p=0.000). Patients with AL required more surgical re-interventions (p=0.000) and one patient in the AL group died of abdominal sepsis and multiorgan failure. These results are similar to those of The LATAM Collaborative Colorectal Surgery Consortium study [63], which analyzed the short-term surgical outcomes of rectal adenocarcinoma treatment in 12 Latin American countries in a multi-center, retrospective manner. They described an overall complication rate of 29.85%, with most of the complications graded >II according to the CDC, an anastomotic leak incidence of 8.9%, and an overall re-operation rate of 9.92% mostly because of AL. They found a 30-day mortality rate of 1.98%.

In the logistic regression model designed to identify risk factors associated with AL, none of the analyzed variables reached statistical significance (p>0.99). Overall, while the model did not identify significant independent predictors, the observed trends underscore the need for further research with larger sample sizes and methodological adjustments. Such efforts could more robustly explore potential associations between clinical variables and the risk of AL.

The principal limitations of the present study are its retrospective nature and the small number of patients. Larger populations may be required to identify these differences, in order to identify a significant association of previously reported risk factors.

## Conclusions

AL remains one of the most severe complications following RC surgery, significantly impacting postoperative morbidity, hospital stay, and the need for surgical reintervention. Although no independent risk factors were identified in the multivariate analysis, we observed that prolonged operative time, increased intraoperative blood loss, and perioperative/postoperative blood transfusion were more frequent among patients who developed AL, suggesting their potential role in anastomotic failure.

The identification of risk factors for AL is essential to improving surgical techniques and perioperative management strategies aimed at reducing its associated morbidity. Our findings underscore the need for careful intraoperative technique, optimization of perioperative hemodynamic status, and early identification of high-risk patients. Future studies with larger cohorts and multicentric data are needed to validate these associations and develop predictive models that guide intraoperative decision-making and improve outcomes in RC surgery.
